# Greenhouse Gas Mass-Balance in Conventional Activated Sludge Wastewater Treatment: A Case Study in Mexico for Developing Countries

**DOI:** 10.1021/acsomega.4c08289

**Published:** 2025-02-06

**Authors:** Pablo Morales-Rico, Jessica Ramos-Díaz, Frédéric Thalasso

**Affiliations:** Biotecnología y Bioingeniería, Centro de Investigación y de Estudios Avanzados del Instituto Politécnico Nacional (Cinvestav), Av. IPN 2508 San Pedro Zacatenco, 07360 Ciudad de México, Mexico

## Abstract

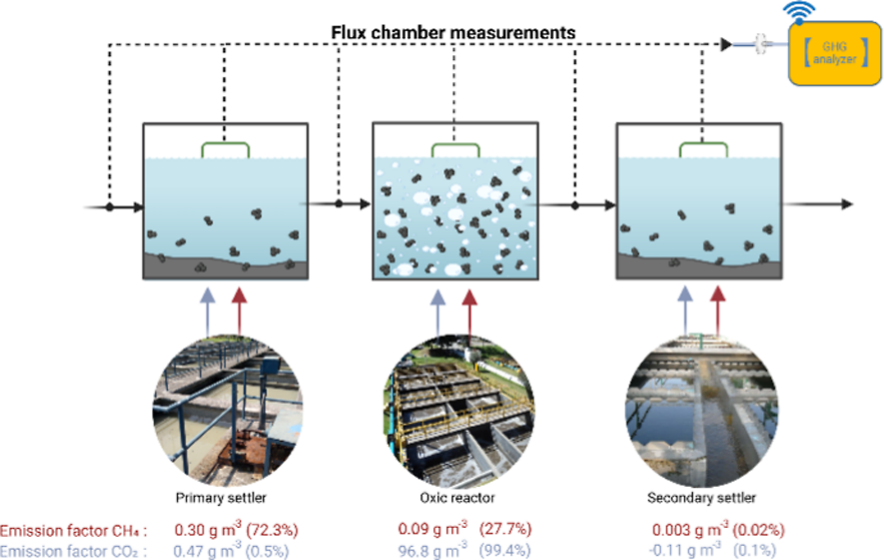

While numerous studies report methane emissions from wastewater treatment plants (WWTPs) in developed countries, few address emissions from plants in developing countries, where outdated technologies, such as the lack of enhanced primary and sludge treatment, are common. Moreover, these studies often rely on indirect calculations rather than direct measurements. Our study fills this gap by providing unit-process-level direct measurements of methane emissions in a conventional WWTP in Mexico, serving as a case study for developing countries. A standard plant was selected and visited on five occasions. It includes a primary settler, an aerated reactor, and a secondary settler, with no sludge treatment in place. Our findings revealed a CH_4_ emission factor of 0.396 ± 0.218 g CH_4_ m^–3^ of treated water, with the primary settler accounting for 72.3 ± 15.9% of emissions, and the aerated reactor contributing 27.7 ± 15.9%. Notably, the emission factors are comparable to those reported for plants with more advanced treatment technologies, suggesting that technological obsolescence may not significantly enhance CH_4_ emissions. Methanotrophy in the aerated reactor was a key process, oxidizing 91–98% of the CH_4_ transported from the primary settler. Additionally, a carbon dioxide (CO_2_) emission factor of 97.4 ± 34.4 g CO_2_ m^–3^ was measured, primarily from the aerated reactor, consistent with the plant’s overall treatment efficiency. These findings provide crucial data for understanding greenhouse gas emissions from WWTPs in developing regions and highlight the need for targeted mitigation strategies.

## 1 Introduction

Among various sources, greenhouse gas (GHG) emissions generated in the waste sector, notably carbon dioxide (CO_2_), methane (CH_4_), and nitrous oxide (N_2_O), significantly contribute to the global GHG budget. Emissions from wastewater treatment plants (WWTPs) play a particularly significant role in this context. The wastewater treatment sector is estimated to be responsible for 5% of global anthropogenic CH_4_ emissions^1^ and 4–5% of global N_2_O emissions.^2^ Despite being quantitatively the major GHG component, CO_2_ emissions are usually excluded from inventories because CO_2_ is the final product of the oxidation of carbon from natural (biogenic) sources, which absorb an equivalent mass of CO_2_. This means CO_2_ emissions do not directly contribute to the increase in its atmospheric concentration.^3^ However, carbon compounds are frequently present in industrial wastewater and should also be considered.^4,5^ In developed countries, most WWTPs combine activated sludge processes with advanced nutrient removal via oxic/anoxic treatment steps and sludge treatment, and their GHG emissions have been relatively well documented.^6−16^ In contrast, simpler WWTPs are used in developing countries such as Mexico, where GHG emissions are far less well-known.^17−23^

For example, in Mexico, stabilization ponds are the most prevalent technology, with 825 facilities, followed closely by conventional activated sludge (CAS) systems, which are used in 787 facilities. However, in terms of flow rate, activated sludge remains by far the predominant treatment technology, handling 103.9 m^3^ s^–1^ or 72% of the total treated wastewater flow rate.^24^ A similar situation is observed in other developing countries in Latin America, as reflected in a study of 2734 WWTPs.^25^ This comprehensive study indicates that activated sludge is the most widely used technology in terms of treated flow rate (58%), followed by stabilization ponds (15%). These activated sludge plants are typically based on a “conventional design”, composed of a primary settler (PS), followed by an aerated reactor and a secondary settler (SS), with sludge recirculation to the reactor. In Mexico, most WWTPs (81%),^26,27^ do not include enhanced primary treatment, and many lack treatment units for the generated sludge.^28^ Consequently, the sludge is often returned to the sewage network or disposed of without treatment. This lack of sludge treatment is a common practice in developing countries.^29,30^ Additionally, the combined handling of industrial and domestic wastewater and the absence of separation between sewage and rainwater further complicate the situation. These factors make activated sludge treatment in developing countries potentially very different in terms of GHG impact compared to advanced treatment schemes in use in developed countries.

Most developing countries are committed to the United Nations Framework Convention on Climate Change, which emphasizes the need to quantify and reduce GHG emissions.^31^ These countries have developed inventories that include emissions from the waste sector, primarily using the TIER 1 methodology of the Intergovernmental Panel on Climate Change.^32^ This basic approach utilizes default emission factors and activity data to estimate GHG emissions,^33−40^ as done, for instance, in conventional WWTPs by El-Fadel and Massoud (2001), Gupta and Singh (2012), Santos et al., (2015), Noyola et al., (2016) Shrestha et al., (2022) Johnson et al. (2022), Ramírez-Melgarejo (2020) and Stringer (2024). Migrating to TIER 2, which involves country-specific emission factors, and TIER 3, which includes comprehensive modeling and direct measurements, would provide more precise and reliable estimates. Some developing countries, such as China, India, and Turkey, are transitioning to higher-tier methodologies.^16,22,41−44^ However, data on GHG emissions measured in the conventional WWTPs prevalent in these nations remain scarce,^42,45−48^ likely hindering the adoption of Tier 2 or 3 methods.

In this context, the present study aimed to provide a detailed analysis of GHG emissions from a full-scale WWTP in Mexico, serving as a case study for developing countries. To achieve this, a GHG mass balance around the PS, aerated reactor, and SS of a conventional treatment plant was conducted on five occasions, with direct measurements of GHG inputs, outputs, and emissions. The primary GHG measured was CH_4_, but CO_2_ was also included, which, although it does not directly add to atmospheric CO_2_ levels, is relevant for assessing wastewater degradation dynamics as the final oxidation product. These results were discussed in the context of organic load degradation to define, for the first time, a locally determined emission factor.

## 2 Material and Methods

### 2.1 Description of the WWTP and Campaigns

**Figure 1:**
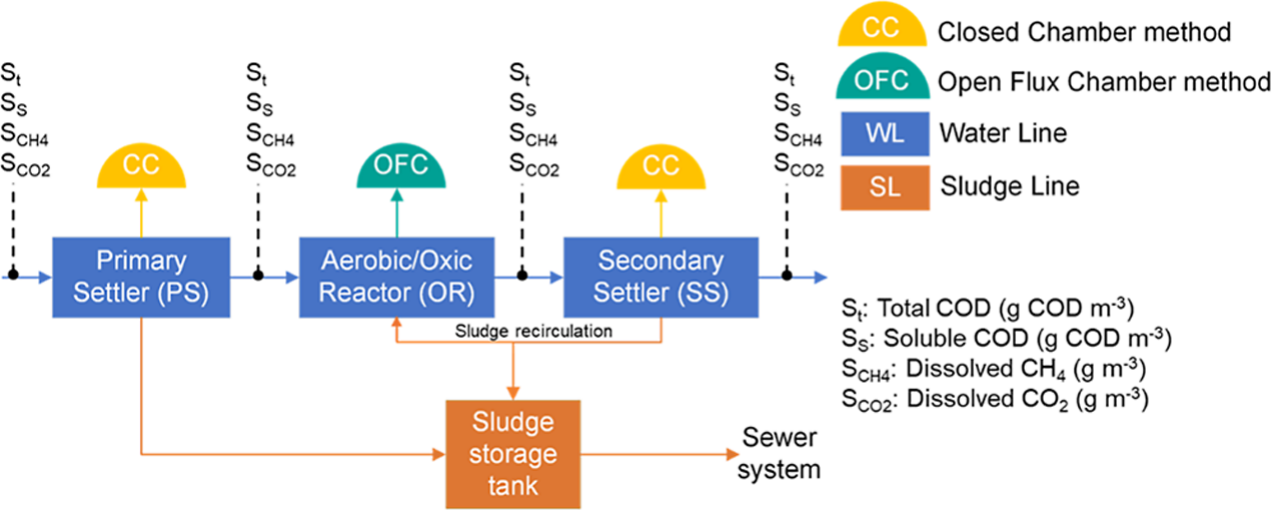
Scheme of the WWTP characterized showing sampling points and methods.

For the purpose of this study, we selected a specific treatment train of a WWTP situated at an undisclosed site within Mexico City (Lat. 19.43; Long. −99.13). It is a CAS plant treating a mean flow rate of about 70 L s^–1^ of urban wastewater with some unidentified industrial effluents. The WWTP follows a common CAS configuration (Figure 1), consisting of a pretreatment unit with a mechanically cleaned coarse screen (manually operated) followed by a PS, an aerobic/oxic reactor (OR) operated with a fine-bubble aeration system and a sludge recirculation system, and a SS. The biological reactor has a plug-flow U-shaped configuration with a total length of 66 m and a total volume of 1562 m^3^. After treatment, the water goes through a final chlorine disinfection unit and is then reused for irrigation purposes. This plant is not equipped with a sludge treatment line; all the sludge produced is partly returned to the aerobic reactor or sent to a storage tank and subsequently discharged into the sewer system (Figure 1). This CAS design is the most common in Mexico City and throughout the country. In Mexico City, there are 22 CAS plants in operation, comprising 84% of the total number of treatment plants.^40^ Nationally, there are 787 CAS facilities, accounting for 28.4% of all WWTPs in Mexico.^24^

The WWTP was visited on five occasions: May 4 and 20, July 29, and August 6 and 24, 2021. These dates were selected to capture both the dry season (May) and the rainy season (July and August), as the rainy season can significantly impact wastewater quality by introducing a dilution effect due to increased water inflow. On each visit, CH_4_ and CO_2_ emissions were measured using floating chambers, and the wastewater along the treatment train was characterized at input and output of each treatment unit. This characterization included dissolved CH_4_ (*S*_CH_4__) and CO_2_ (*S*_CO_2__) concentrations, total Chemical Oxygen Demand (COD; *S*_t_) determined from unfiltered water samples, and soluble COD (*S*_s_), determined filtering the samples with 0.45 μm pore filter,^49^ according to the protocol reported by van Loosdrecht et al., 2016.

### 2.2 CH_4_/CO_2_ Emissions

Greenhouse gas emissions (CH_4_ and CO_2_) were measured in each process unit. In both the primary and SSs, with no aeration, GHG emissions were measured using a floating static chamber method at the center of the units and along 7 equidistant longitudinal locations. The chamber was constructed from a plastic basin painted white to reflect light, with a floating noodle attached to its perimeter to ensure buoyancy (contact area with water: 0.1385 m^2^; headspace volume: 14 L). The chamber headspace was connected to an ultraportable greenhouse gas analyzer (UGGA, Los Gatos Research, USA) in a closed-loop setup. Continuous measurements of CH_4_ and CO_2_ were taken for 5 min, excluding the first 30 s after positioning the chamber on the water surface. Each flux was measured in triplicate, calculated from the slope of the gas concentration multiplied by the chamber headspace volume and divided by its contact area. In the aerated reactor, CH_4_ and CO_2_ emissions were measured at the center of the reactor along 7 equidistant longitudinal locations using an open flux chamber method^50^ previously described by Morales-Rico et al. (2024). Briefly, this method used the same GHG analyzer as the static chamber method (UGGA, Los Gatos Research, USA) and a specially designed floating chamber (referred to as the “A″ chamber in Morales-Rico et al., 2024). This chamber is placed at the desired location on the water surface, where the gas emitted from the water surface mixes with the chamber headspace, with excess gas being evacuated through a vent. As a result, the chamber headspace, initially filled with atmospheric air, is gradually replaced by the gas emitted from the water, eventually reaching a new steady state. By adjusting the chamber’s mass balance based on the measured trends of CH_4_ and CO_2_ concentrations, the gas residence time (dependent on the gas flow rate) and the concentration of the emitted gas are determined. This allowed for the calculation of the flux as the product of the gas flow rate and the CH_4_/CO_2_ gas concentration at steady-state divided by the area of the chamber deployed. Each measurement was done in triplicate, over 5 min of deployment time.

### 2.3 Dissolved CH_4_/CO_2_ Concentration

Dissolved CH_4_ (*S*_CH_4__) and CO_2_ (*S*_CO_2__) concentrations at the influent and effluent of each process unit (Figure 1) were determined in triplicate on each date using a discrete headspace equilibration method. This method involved taking a sample of water in a 60 mL plastic syringe, ensuring the absence of air bubbles. Then, 20 mL of water was gently evacuated and replaced with CH_4_- and CO_2_-free nitrogen (99.999% N_2_, Praxair, Mexico). The syringe content was vigorously shaken for 30 s for equilibration, and the water was evacuated. Next, 10 mL of the remaining headspace gas was injected into a continuous flow of nitrogen connected to the UGGA in an open circuit. The presence of CH_4_ and CO_2_ in the gas sample was detected as a peak response, which was integrated after proper calibration with standard CH_4_/CO_2_ samples. Finally, *S*_CH_4__ and *S*_CO_2__ in the liquid subsamples were derived from Henry’s solubility constant.^51^ This process included syringe calibration and careful consideration of water temperature, and the volumes of gas and water involved in each step. Unlike standard headspace equilibration methods, we did not use a microbial inhibitor because the interval between water sampling and analysis was typically less than 5 min. This brief time frame minimizes the potential for microbial activity to alter the sample’s composition.

### 2.4 Physicochemical Parameters

To determine the wastewater composition through the treatment units, total chemical oxygen demand (COD) (*S*_t_) and soluble COD (*S*_s_) was determined in triplicate from the influent and effluent of each process unit, on each date. In the present work, soluble COD was considered as the readily biodegradable fraction of the COD.^52^ For the determination of *S*_t_, thoroughly mixed wastewater samples were directly analyzed, while for *S*_S_, samples were centrifuged at 7000 rpm for 10 min (K2015, Centurion Scientific, Mexico), and the supernatant was filtered through a 0.45 μm glass fiber filter according to the protocol previously reported.^49^ An appropriate volume of each sample (2–10 mL) was transferred to COD low and middle range kit digestion vials (Hanna Instruments, Mexico) containing digestion reagents. The vials were then heated at 150 °C for 2 h to ensure complete oxidation of the organic matter. After cooling, the absorbance of the digested samples was measured using a spectrophotometer (Genesys 20, Thermo Spectronic, Mexico) at 600 nm. Quality control included blanks, triplicates, and standard reference materials to ensure accuracy and precision. In addition to COD, a multiparametric probe (HI9829, Hanna Instruments, Mexico) was used to measure dissolved oxygen (DO) concentration, pH, oxidation–reduction potential (ORP), and water temperature.

### 2.5 Mass Balance

The CH_4_ and CO_2_ mass balance over the treatment units was established by considering only the dissolved CH_4_/CO_2_ input and output, emissions to the atmosphere, and a biological component expressed as the net production rate. For CH_4_, this net production rate can be positive in the case of net methanogenesis or negative in the case of net methanotrophy (CH_4_ oxidation).
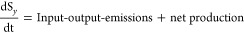
1
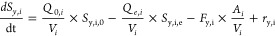
2

Where *S*_*y*_ is the concentration of compound *y* (CH_4_ or CO_2_) in the water phase of unit *i*, *Q*_0,*i*_ and *Q*_*e*,*i*_ are the influent and effluent flow rates (m^3^ h^–1^), respectively, at which the treatment unit is operated, *V*_*i*_ is the volume of process unit (m^3^), *A*_*i*_ is the surface area of the process unit, *S*_*y*,*i*,0_ and *S*_*y*,*i*,*e*_ are the influent and effluent concentrations of compound *y* (g m^–3^), respectively, *F*_*y*,*i*_ is the flux of the compound *y* emitted to the atmosphere (g m^–2^ h^–1^), and *r*_*y*,*i*_ is the net production rate of compound *y* (g m^–3^ h^–1^).

Assuming steady-state conditions, where the concentration does not change over time, and mass conservation, meaning that the influent and effluent flow rates are equal, eq 2 can be written as
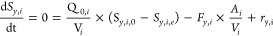
3

In eq 3, each term is expressed as the mass of the compound of interest per unit volume of the process unit and per unit of time. Alternative units, commonly used in the literature, consider each term as the mass of compound *y* per unit volume of water treated. For this purpose, eq 3 was modified by multiplying each term by *V*_*i*_/*Q*_0,*i*_, as follows
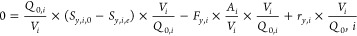
4
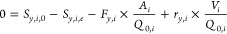
5

It is worth noting that eq 5 can be applied to a specific treatment unit or over an entire treatment train, which in this case is written as follows
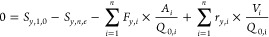
6Where *S*_*y*,1,0_ is the concentration of compound *y* (CH_4_ or CO_2_) in the influent of the first unit of the treatment train, *S*_*y*,*n*,0_ is the concentration of the same compound in effluent of the last unit, and *n* is the number of units included in the treatment train. The third term in eq 6, is the Emission factor (EF) widely reported in the field.
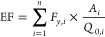
7

It is important to underline that, in this study, the effluent from one treatment unit was assumed to be equal to the influent of the succeeding unit. This approach was supported by two considerations. First, the residence time within the pipes connecting the units was considered very short compared to the in-unit residence time and was therefore assumed to have a negligible impact on water composition and CH_4_/CO_2_ concentrations. Second, unlike the primary and SSs, where inflow and outflow were clearly identifiable, the design of the biological reactor did not allow for direct sampling, as both the inflow and outflow points were located below the water level, making sampling impossible.

### 2.6 Statistical Analysis

To accurately estimate the uncertainty linked to combined parameters (added, multiplied or divided), we applied standard error propagation techniques based on the nature of the mathematical operations involved.^53,54^ These methods ensured that the propagated errors in the final parameters were accurately quantified, considering the uncertainties of the individual measurements. Statistical comparisons among results were determined according to Tukey’s honest significance test. Most variables had a positive skew, which were log-transformed to achieve the normality of the ANOVA residuals. This analysis was performed with Origin(Pro) software (Version 2016, Northhampton, USA).

## 3 Results and Discussion

### 3.1 Plant Operation

During the sampling period, The influent COD varies seasonally, with an average of *S*_t_ of 0.341 ± 13% kg COD m^–3^. The interquartile range spans from 0.28 to 0.36 kg COD m^–3^, with 50% of the data falling within this range. This variability is attributed to the dilution factor prevalent during the rainy season, as Mexico City lacks a separate rainwater drainage system. This dilution factor has a greater impact on the soluble COD (*S*_s_), as evidenced by a measured variation of 0.24 ± 20% kg COD m^–3^. The overall removal efficiency during the visits, in terms of COD removal, was 84.4 ± 7%. We also measured the physicochemical parameters of the water for each process unit, including pH, DO concentration, ORP, and temperature. Briefly, the PS was characterized by strict anaerobic conditions, evidenced by undetectable levels of DO, and by a mean ORP of −204.3 ± 82 mV. We hypothesize that these anaerobic conditions resulted from the combination of no aeration, relatively still water, and the high organic load promoting dissolved oxygen uptake through microbial respiration. The mean pH and temperature of the PS were 7.05 ± 0.12 and 26.5 ± 1.9 °C, respectively. Contrastingly, the aerated reactor was operated under aerobic conditions, with a mean DO concentration of 1.21 ± 0.44 mg L^–1^, and an ORP of 101.3 ± 29 mV. The mean pH and temperature of the aerated reactor were 7.9 ± 17 and 22.07 ± 1.15 °C, respectively. Lastly, the SS was suboxic, with a mean DO concentration of 0.18 ± 0.13 mg/L, and an ORP of −37.3 ± 18 mV. The mean pH and temperature of the SS were 7.87 ± 0.20 and 22.8 ± 0.82 °C, respectively.

### 3.2 CH_4_ Mass Balance

**Figure 2:**
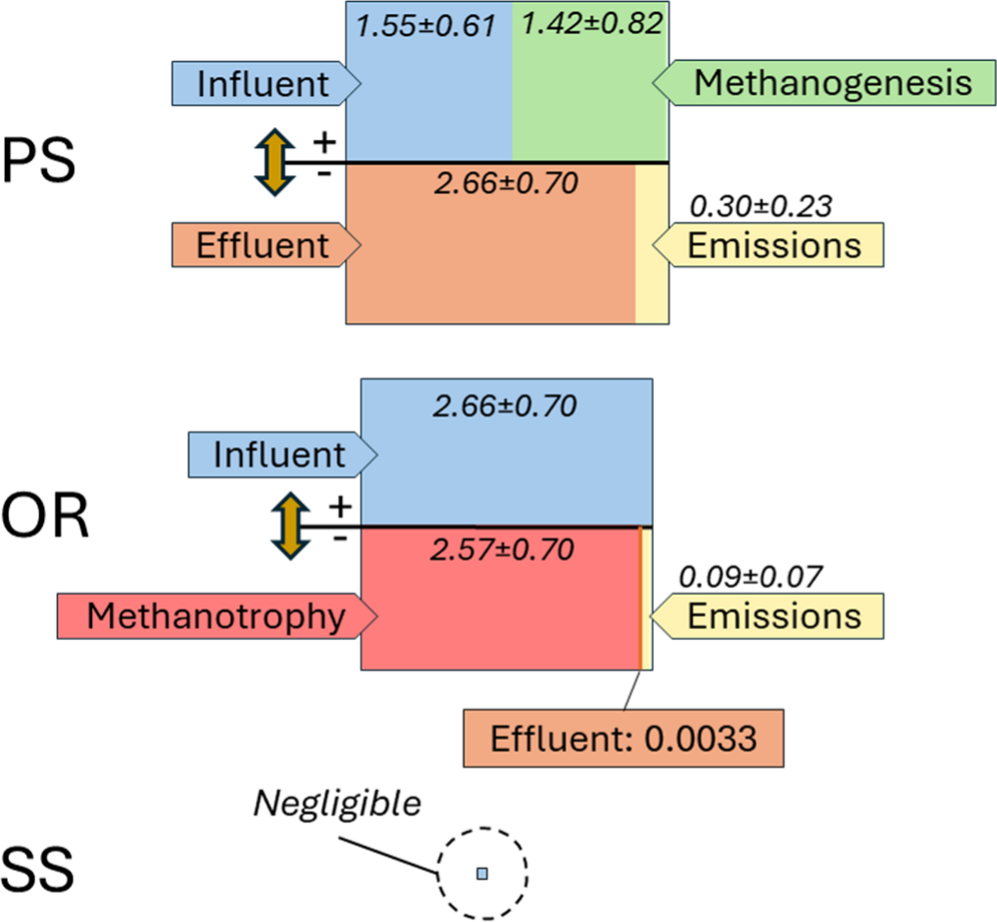
Mass balance of CH_4_ depicted using divided boxes. The upper section of each box represents the positive inputs, while the lower section depicts the negative outputs. Mass balances in the PS, the OR, and the SS are shown. All values are expressed in mass per unit of wastewater volume treated (g m^–3^). The size of the boxes is proportional to the magnitude of the processes involved.

As a result of anaerobic conditions and relatively high input of organic matter, the PS operated as a CH_4_ production unit, producing 1.42 ± 0.82 g CH_4_ m^–3^ of treated water. This production was slightly lower than the CH_4_ input (*S*_CH_4__), which was 1.55 ± 0.61 g m^–3^. Thus, CH_4_ produced in the sewer system prior to entering the treatment plant was the main source of methane to the PS. As a result of this relatively high *S*_CH_4__, the settler emitted CH_4_ at a flux of 10.2 ± 7.6 g m^–2^ d^–1^, corresponding to an emission factor of 0.30 ± 0.23 g m^–3^ of treated water or 3.71 ± 2.66 × 10^–3^ kg CH_4_ kg^–1^ COD removed. These CH_4_ emissions represent a relatively minor component of the CH_4_ mass balance, constituting approximately 19% of the influent *S*_CH_4__ and 11% of the effluent *S*_CH_4__ (Figure 2). In the OR, where measurements indicated uniform aerobic conditions and where CH_4_ was entering at a moderate concentration of at 2.66 ± 0.70 g m^–3^, the effluent contained only trace levels of *S*_CH_4__ at 3 × 10^–3^ g m^–3^. We hypothesized that the drastic decrease in *S*_CH_4__ was primarily caused by emissions to the atmosphere due to air stripping, with a minor contribution from aerobic CH_4_ oxidation (methanotrophy). This hypothesis was put in doubt by CH_4_ flux measurements, which turned out to be lower than anticipated. In fact, we found that the CH_4_ emissions were relatively small compared to those observed in the PS, at 1.36 ± 0.98 g m^–2^ d^–1^, corresponding to an emission factor of 0.09 ± 0.07 g CH_4_ m^–3^ of treated water or 4.15 ± 2.51 × 10^–4^ kg CH_4_ kg^–1^ COD removed (Figure 2). This CH_4_ emission was only about 3.4% of the influent *S*_CH_4__. This relatively small contribution of CH_4_ emissions suggests that most of the CH_4_ (range 91–98%, mean 96.3 ± 2.8%) was oxidized within the reactor, suggesting that methanotrophy was a dominant process, controlling the CH_4_ mass balance in the WWTP. This is in accordance with the literature that shows that most of the dissolved CH_4_ is oxidized in aerated reactors, at a proportion that depend on the operating conditions of the reactor.^7,55,56^ Lastly, after characterizing the PS and the aerobic reactor, we characterized the SS. The SS was confirmed as a minor process unit, in terms of CH_4_, with emissions of 1.44 ± 1.54 × 10^–3^ g m^–2^ d^–1^, i.e., more than 3 orders of magnitude lower than emissions observed in the PS and the aerobic reactor.

Globally, the CH_4_ emission factor for the entire treatment plant was 0.396 ± 0.218 g CH_4_ m^–3^ of treated water or 1.39 ± 0.74 × 10^–3^ kg CH_4_ kg^–1^ COD removed, of which 72.3 ± 15.9% was originating from the PS and 27.7 ± 15.9% from the aerated reactor. This emission factor is within the range previously reported in the literature, as depicted in Table 1. It is worth noting that data reported in Table 1 are all measured data (excluding other determination methods), and are exclusively those reported for the activated sludge process, i.e. excluding sludge digestion and treatment. The range of emission factors previously reported cover several orders of magnitude, with an interquartile range of 0.12–1.56 g CH_4_ m^–3^ of treated water and a median of 0.42 g CH_4_ m^–3^, relatively close to the mean value determined in the present study. Regarding the conversion to CH_4_ (expressed as COD) of the removed COD, we observed a very small conversion yield, at 0.55 ± 0.38% over the entire operation of the WWTP.

**Table 1:** CH_4_ EFs Reported for CAS Technology in the Literature and This Work

WWTP	country	EF (g m^–3^)	references
this work	Mexico	0.39	
Chongqing region	China	11.6	Wang et al., 2022^22^
Jinan	China	0.16	Wang et al., 2022^22^
Viikinmäki^a^	Finland	2.9	Mölsä., 2020^19^
Bellheim	Germany	0.5	Tumendelger et al., 2019^2^
Akiu	Japan	0.42	Masuda et al., 2018^14^
Jungryang	Korea	1.03 × 10^–5^	Hwang et al., 2016^13^
Cerro de la Estrella	Mexico	6.4	Noyola et al., 2018^47^
Avedøre	Norway	0.12	Faragò et al., 2022^21^
Granollers	Spain	0.06	Rodriguez-Caballero et al., 2014^8^
WWTP in Galicia	Spain	8.05 × 10^–2^	Flores et al., 2021^20^
Linköping	Sweden	0.48	Gålfalk et al., 2022^63^
Källby	Sweden	0.21	Delre et al., 2019^23^
Papendrecht	The Netherlands	2.44	Daelman et al., 2012^7^
Kortenoord	The Netherlands	1.56	Daelman et al., 2012^7^
Durham	USA	0.14	Czepiel et al., 1993^17^
median value		0.42	
min		1.03 × 10^–5^	
max		11.60	

a Receives industrial wastewater.

### 3.3 CO_2_ Mass Balance

**Figure 3:**
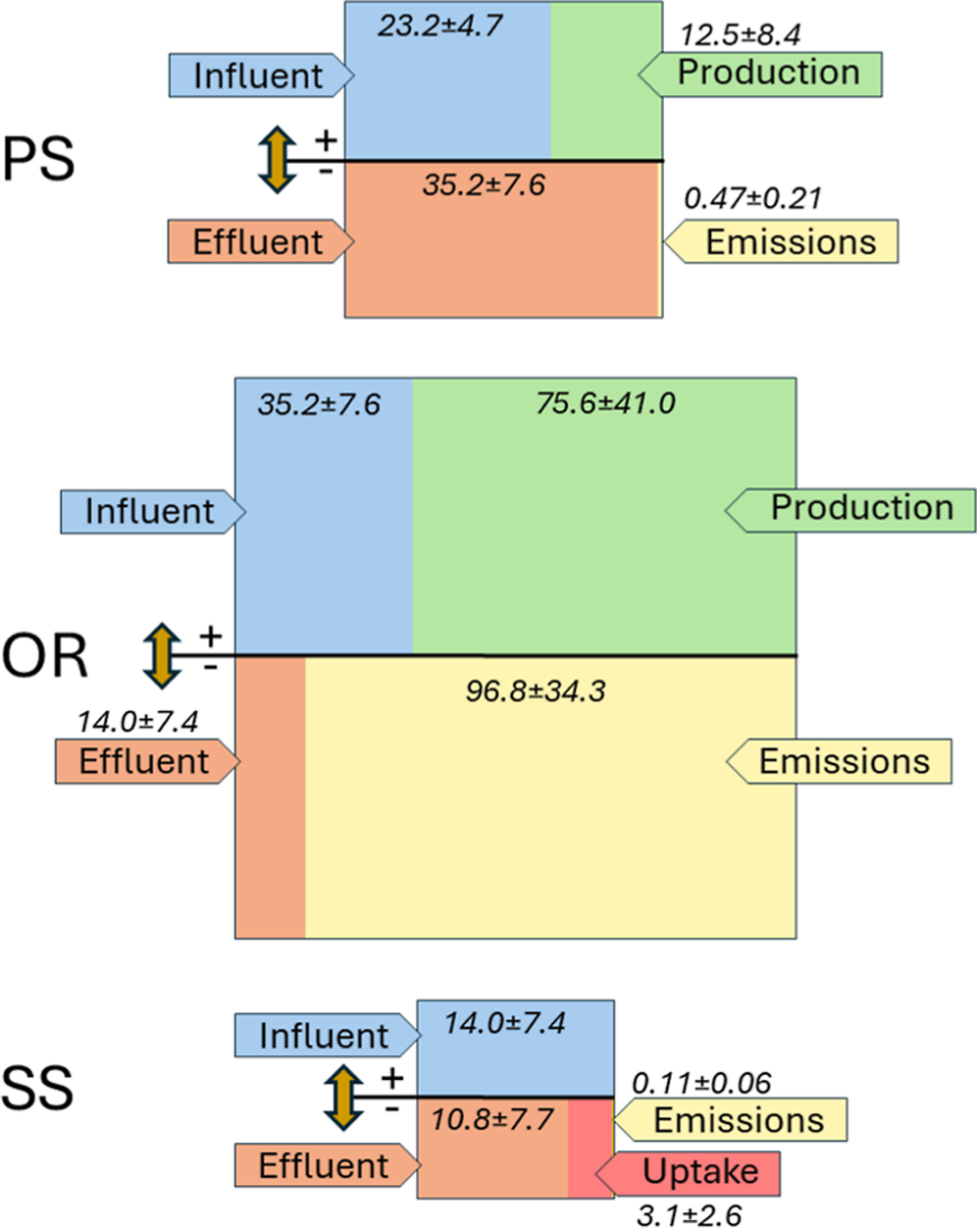
Mass balance of CO_2_ depicted using divided boxes. The upper section of each box represents the positive inputs, while the lower section depicts the negative outputs. Mass balances in the PS, the OR, and the SS are shown. All values are expressed in mass per unit of wastewater volume treated (g m^–3^). The size of the boxes is proportional to the magnitude of the processes involved, though not to the same scale as those in Figure 2.

Similarly to what was observed for CH_4_, CO_2_ accumulation was detected in the PS, as the wastewater exited the treatment unit with a concentration 51.7% higher than its influent concentration. The observed accumulation reached 12.5 ± 8.4 g m^–3^ of treated wastewater (Figure 3) and was only minimally offset by emissions to the atmosphere. These emissions corresponded to an emission factor of 0.47 ± 0.21 g m^–3^, accounting for just 3.7% of the accumulated CO_2_. This limited emission could be attributed to the still nature of the water surface in the PS, which is unfavorable for gas transfer to the atmosphere. Notably, CO_2_ production in the PS was about ten times higher than CH_4_ production, with a corresponding mass ratio of 1:10.

In the aerated reactor, significant CO_2_ production was also observed, with high variability ranging from 38.3 to 138.7 g m^–3^ of treated water and a mean of 75.6 ± 41.0 g m^–3^ (Figure 3). This production level can reasonably be associated with respiratory processes, stoichiometrically balanced with COD removal, as discussed later in this section. Due to gas stripping and turbulence at the reactor surface caused by aeration, CO_2_ emissions were a dominant process, averaging 96.8 ± 34.3 g m^–3^. These emissions accounted for 87.4% of the combined influent and produced CO_2_, meaning only 12.6% of the CO_2_ entering the reactor or produced within it was released through the reactor effluent. The CO_2_ emission factor of 96.8 g m^–3^ corresponded to 0.26 ± 0.15 kg CH_4_ kg^–1^ COD removed, which is approximately 3 orders of magnitude higher than that of CH_4_, with 32% originating from influent dissolved CO_2_ and 68% produced within the reactor.

Unlike CH_4_, the CO_2_ mass balance in the SS was not negligible but still exhibited significant uncertainty. No significant difference was observed between the influent and effluent CO_2_ concentrations, and the CO_2_ flux was relatively small (0.11 ± 0.02 g m^–3^), preventing definitive conclusions about whether the SS exhibited significant CO_2_ uptake or production.

Globally, the CO_2_ emission factor for the entire treatment plant was 97.4 ± 34.4 g CO_2_ m^–3^ of treated water or 0.34 ± 0.12 kg CO_2_ kg^–1^ COD removed, of which 99.4 ± 0.3% originated from the aerated reactor, with negligible amounts emitted from both the primary and SSs. From eq 6, the total CO_2_ production in the treatment train (sum of outputs less inputs) was estimated to be 84.9 ± 36.7 g CO_2_ m^–3^. In the aerated reactor, the CO_2_ production was 75.6 ± 41.0 g CO_2_ m^–3^, while the COD removal was 230.2 ± 53.4 g COD m^–3^. This allows for the determination of the ratio of CO_2_ produced to COD removed, which was 0.135 to 0.523 g CO_2_ g^–1^ COD in the aerated reactor. Assuming a wastewater and biomass mean composition of C_18_H_19_NO_9_,^57^ the process equation which is in agreement with the mean measured CO_2_/COD ratio is described by
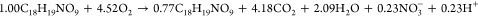
8

The measured CO_2_/COD ratio corresponded to a biomass growth yield of 0.442–0.635 g of biomass growth per g of COD removed, with a mean of 0.539 g g^–1^. This range aligns with values suggested for domestic wastewater treated by activated sludge.^55^

### 3.4 Greenhouse Gas Balance

When combining CO_2_ and CH_4_ emissions in terms of global warming potential (GWP), the total emission factor of the treatment train, assuming a GWP of 30 for CH_4_ over a 100 year time frame,^58^ was 109.3 ± 35.1 g CO_2_ equivalents m^–3^ of treated water, with CH_4_ contributing 10.82% of the total emissions. It is important to recall that, in this study, CO_2_ was considered the final product of the oxidation of carbon from natural (biogenic) sources, which absorb an equivalent mass of CO_2_ and therefore do not directly contribute to increased atmospheric CO_2_ concentrations. Based on this consideration, anthropogenic emissions were limited to CH_4_, with a total emission factor of 11.85 g CO_2_ -eq m^–3^ of treated water. It should be acknowledged, however, that this approach excludes fossil fuel-derived carbon compounds, which are often present in wastewater and should be quantified as part of anthropogenic CO_2_ emissions.

Importantly, this study did not account either for CH_4_ emissions associated with the sludge produced during the activated sludge treatment process, which is often discharged directly into the sewer system without further treatment—a widespread practice in developing countries. Consequently, the potential CH_4_ emissions from the anaerobic decomposition of this sludge within the sewer network have not been accounted for in our analysis. Anaerobic conditions in sewers can lead to significant methane production^59−61^ suggesting that CH_4_ production from discharged sludge could potentially be a major component of the overall emission factor. Future studies should aim to incorporate the entire lifecycle of sludge handling, including its fate postdischarge, to provide a more comprehensive assessment of GHG emissions from wastewater treatment processes. However, this is a challenging task due to the diversity of conditions encountered in complex sewage networks and the difficulties associated with measuring CH_4_ emissions in situ or replicating these conditions in a model study.^62^

## 4 Conclusions

This study presents a pioneering analysis of CH_4_ and CO_2_ emissions from a CAS WWTP in Mexico, employing a Tier 3-equivalent approach with process unit-level resolution. The CH_4_ emission factor for the entire treatment plant was 0.396 ± 0.218 g CH_4_ m^–3^ of treated water or 1.39 ± 0.74 × 10^–3^ kg CH_4_ kg^–1^ COD removed, primarily originating from the PS. This emission factor falls within the range reported for plants with more advanced treatment technologies. While CO_2_ emissions are rarely included in inventories, they were considered in this study, with the mean CO_2_ emission factor for the entire treatment plant estimated at 97.4 ± 34.4 g CO_2_ m^–3^ of treated water or 0.34 ± 0.12 kg CO_2_ kg^–1^ COD removed. Unlike CH_4_, 99.4 ± 0.3% of CO_2_ emissions originated from the aerated reactor, consistent with the observed COD removal efficiency. These results suggest that the simple technology commonly used in Mexico and other developing countries—comprising a PS, aerated reactor, and SS—does not inherently produce higher GHG emissions compared to more sophisticated systems. However, this conclusion does not account for the absence of sludge treatment, a prevalent practice in developing countries, nor the potential CH_4_ emissions from untreated sludge discharged into the sewage system. Transitioning to Tier 2 and Tier 3 methodologies, which provide more precise and reliable GHG estimates, should therefore include the handling and fate of sludge postdischarge. Similarly, the present study did not consider fossil carbon which is often introduced through fossil fuel-derived compounds in wastewater, which should also be accounted for in future analyses. Both aspects are critical for improving the accuracy of GHG inventories and aligning with the commitments of developing countries under the United Nations Framework Convention on Climate Change.

## Abbreviations

CAS: conventional activated sludge
EF: emission factor
GHG: greenhouse gas
PS: primary settler
OR: aerobic/oxic reactor
WWTP: wastewater treatment plant
